# Can visual analogue scale be used in radiologic subjective image quality assessment?

**DOI:** 10.1007/s00247-018-4187-8

**Published:** 2018-07-04

**Authors:** Kathrine Rydén Suther, Einar Hopp, Bjarne Smevik, Arnt Eltvedt Fiane, Harald Lauritz Lindberg, Stig Larsen, Charlotte de Lange

**Affiliations:** 1 Division of Radiology and Nuclear Medicine, Oslo University Hospital, Rikshospitalet, P.O. Box 4950, Nydalen, 0424 Oslo, Norway; 2 Faculty of Medicine, University of Oslo, Oslo, Norway; 3 Department of Cardiothoracic Surgery, Oslo University Hospital, Rikshospitalet, Oslo, Norway; 4 Faculty of Veterinary Medicine, Centre for Epidemiology and Biostatistics, Norwegian University of Life Sciences, Oslo, Norway

**Keywords:** Adolescents, Coronary artery, Fixed point scale, Heart, Image quality, Magnetic resonance imaging, Visual analogue scale

## Abstract

**Background:**

Assessment of qualitative or subjective image quality in radiology is traditionally performed using a fixed-point scale even though reproducibility has proved challenging.

**Objective:**

Image quality of 3-T coronary magnetic resonance (MR) angiography was evaluated using three scoring methods, hypothesizing that a continuous scoring scale like visual analogue scale would improve the assessment.

**Materials and methods:**

Adolescents corrected for transposition of the great arteries with arterial switch operation, ages 9–15 years *(n*=12), and healthy, age-matched controls (*n*=12), were examined with 3-D steady-state free precession magnetic resonance imaging. Image quality of the coronary artery origin was evaluated by using a fixed-point scale (1–4), visual analogue scale of 10 cm and a visual analogue scale with reference points (figurative visual analogue scale). Satisfactory image quality was set to a fixed-point scale 3=visual analogue scale/figurative visual analogue scale 6.6 cm. Statistical analysis was performed using Cohen kappa coefficient and agreement index.

**Results:**

The mean interobserver scores for the fixed-point scale, visual analogue scale and figurative visual analogue scale were, respectively, in the left main stem 2.8, 5.7, 7.0; left anterior descending artery 2.8, 4.7, 6.6; circumflex artery 2.5, 4.5, 6.2, and right coronary artery 3.2, 6.3, 7.7. Scoring with a fixed-point scale gave an intraobserver κ of 0.52–0.77 while interobserver κ was lacking. For visual analogue scale and figurative visual analogue scale, intraobserver agreement indices were, respectively, 0.08–0.58 and 0.43–0.71 and interobserver agreement indices were up to 0.5 and 0.65, respectively.

**Conclusion:**

Qualitative image quality evaluation with coronary 3-D steady-state free precession MR angiography, using a visual analogue scale with reference points, had better reproducibility compared to a fixed-point scale and visual analogue scale. Image quality, being a continuum, may be better determined by this method.

## Introduction

A key factor in evaluating and finding the best imaging technique is an accurate and reproducible validation method of image quality assessment. Guidelines for image quality criteria have been established for several ionizing techniques, such as radiography and computed tomography (CT) of the thorax [1–3] and proposed for cardiac magnetic resonance imaging (MRI) [4]. However, these guidelines mainly focus on the objective measurements of image quality. Subjective or qualitative image quality criteria are mentioned, but little is reported about which method is recommended for the evaluation.

There are three main approaches proposed to evaluate subjective image quality -- the fixed-point scale and the visual analogue scale without and with reference marks.

Subjective image quality assessment has commonly been performed using an ordinal fixed-point scale. This is true for different radiologic methods, including conventional radiography, CT, MRI and also for coronary magnetic resonance angiography (MR angiography) [4–9]. A fixed-point scale is usually compared to a Likert scale where entities are ordered according to quantitative features, often in 5 points ranging from “totally disagree” to “totally agree” [10]. In various older and newer publications, the scales vary from 3 up to 5 points, in general. The 3- and 5-point ordinal scales, from a statistical point of view, have the disadvantage of a non-normal non-parametric distribution. The central measure of these scales is not the parametric mean, but the geometric mean or median.

A visual analogue scale is a psychometric response scale that can be used in questionnaires. It notes subjective characteristics or attitudes that cannot be directly measured. It is basically a horizontal line on which an observer indicates his or her response by making a mark. A visual analogue scale presented as 10-cm ruler is a documented method for scoring continuous soft data like pain and mood [10–12].

For sample size calculations and full-scale variance, a visual analogue scale could be a better option to determine image quality. This method has also been used for visual grading of endoscopic images of gastric lesions [13–15]. Only a few recent radiologic studies have used a visual analogue scale for subjective image quality evaluation [16–18]. Stengel and co-workers [16] used a 10-cm visual analogue scale to assess whole-body CT protocols and revealed in their pilot study with two observers, an arithmetic mean of the raters´ scores with pooled standard deviation and with little difference in image quality. However, they did not perform an observer agreement analysis [16]. The two other studies did not report interobserver variability in their visual analogue scale scoring, but Papanikolaou et al. found that the observers´ experience influenced the scoring [17, 18]. The only study we have found comparing a visual analogue scale to a fixed-point scale showed equal performance of the scoring methods with a preference for the visual analogue scale, but this study was evaluating endoscopic images of erosive mucosal lesions [19].

Using a modified visual analogue scale adding reference points or text to the 10-cm scale, a figurative visual analogue scale, could theoretically improve discrimination of the score and be a more specific and reproducible scoring method. This has been proposed in self-evaluation of pain [11] and again used in one endoscopic study that found adding a reference text was well suited for gradual evaluation of mucosal findings (visual analogue scale versus figurative visual analogue scale), but may lead to a tendency to accumulate scores around these points approximating a fixed-point scale situation [15].

In children and adults with congenital heart disease, cardiac imaging follow-up is required throughout life and cardiovascular MRI, being a nonionizing technique, is mainly recommended over conventional angiography and CT [20]. In transposition of the great arteries, there is a ventricular-arterial discordance, where the aorta and the pulmonary artery have switched places. An arterial switch operation is performed in the early neonatal period, where the great arteries are switched and the coronary arteries reimplanted. Late postoperative coronary artery events have been reported, and regular follow-up for coronary artery patency is recommended [21–23]. Conventional coronary angiography is considered the gold standard for assessing coronary artery patency. However, coronary MR angiography techniques have been developed with improved performance also at high field strength MRI units and could be an attractive alternative if sufficient image quality could be documented [24–27].

In this study, we aimed to investigate the performance of three different methods for assessing qualitative image quality of 3-T coronary MR angiography without contrast enhancement. We hypothesize that using figurative visual analogue scale, a continuous scoring method with predefined reference points, would give a more robust image quality assessment compared to fixed-point scale and visual analogue scale.

## Materials and methods

The study was approved by the local ethics committee on human research, and all subjects and their parents/caretakers gave their written, informed consent to participation. All procedures performed in studies involving human participants were in accordance with the ethical standards of the institutional and/or national research committee and with the 1964 Helsinki declaration and its later amendments or comparable ethical standards.

### Subjects

Patients ages 9–15 years, who had undergone surgical correction for transposition of the great arteries in the neonatal period at our university hospital, were invited to a larger prospective study. Twelve randomly chosen patients, two from each age cohort (same birth year) were registered and underwent coronary MR angiography with steady-state free precession (SSFP). Twelve healthy, age-correlated individuals also underwent the same sequence. MRI was performed without general anaesthesia or sedation.

### MRI protocol

Examinations were performed on a 3-T Skyra MRI system (Siemens Medical Solutions, Erlangen, Germany) unit. A coronal 3-D whole-heart, fat-saturated, respiratory-gated and electrocardiogram-triggered balanced SSFP sequence covering the thoracic cage was done, with the following imaging parameters: TR/TE=240 ms/1.31 ms, flip angle 90°, no magnetization preparation pulse, bandwidth 1502 Hz/pixel, field of view 350 mm, matrix 208 × 187, and reconstructed voxel size 0.8 × 0.8 × 1.0 mm^3^.

### Data analysis and scoring

Post-processing of the images was performed offline at a Vitrea work station (Toshiba Medical Systems, Tokyo, Japan). To display the origin and proximal parts of the reimplanted coronary arteries, standardized multiplanar reconstructions were created from the coronal SSFP sequence. The left main stem, the left anterior descending artery, the circumflex and the right coronary artery as well as possible coronary anomalies were evaluated.

Evaluation of subjective image quality was performed blinded by two radiologists, KRS and CdL, with, respectively, 3 years and 10 years of experience reading cardiac MRI in congenital heart disease. Scoring was performed in a standardized, blinded fashion for both the intra- and interobserver evaluation with three scoring systems. Intra-observer scoring was performed with 1–2 months’ interval to avoid recognition bias. As a preparation, a joint reading of a few cases, where the readers agreed on the different scores in consensus, was performed.

Three different scoring methods to evaluate each image set were used -- a fixed-point scale, a visual analogue scale and a figurative visual analogue scale (figurative visual analogue scale).

The fixed-point scale had the following scores: 1=not possible to interpret/poor; 2=moderate, 3=good and 4=excellent, with the image criteria described in detail in Table 1.

**Table 1:** Scoring criteria for fixed-point, visual analogue and figurative visual analogue scales

Fixed-point scale score	Visual analogue/ figurative visual analogue scale score	Criteria
1	0 cm	Ostium not visible/barely visible with highly blurred vessel wall
2	3.3 cm	Visible ostium with moderately blurred vessel wall
3	6.6 cm	Ostium with slightly blurred vessel wall
4	10 cm	Ostium with excellent sharply defined vessel wall with a quality comparable to CT coronary angiography

The visual analogue scale was performed using a two-sided ruler with a 10-cm-long line without markings with the absolute minimum and maximum scores at the extremities on one side of the ruler, and the same 10-cm-long line with cm marks on the back. Along this line is a sliding marker showing the same spot on the 10-cm line on both sides of the ruler. The recording was done by making one point with this sliding marker on the plain side of the ruler, and then turning the ruler to find the score on the 10-cm marked side.

**Fig. 1:**
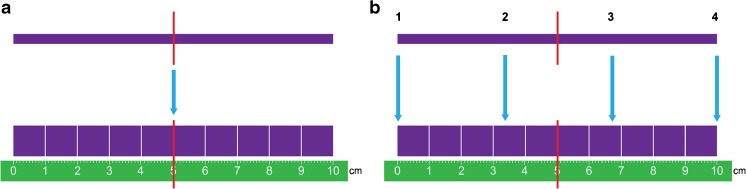
Two continuous methods for qualitative image quality assessment. **a** Visual analogue scale with the plain side with the slider (*red marker*) uppermost and the corresponding 10-cm ruler on the back lowermost. **b** Figurative visual analogue scale with reference marks at 0, 3.3, 6.6 and 10 cm on the plain side uppermost and the corresponding 10-cm ruler on the back lowermost

Consequently, corresponding to a fixed-point scale, 0 cm equals fixed-point scale score 1 and 10 cm equals fixed-point scale score 4 (Table 1 and Fig. 1).

The figurative visual analogue scale uses the same procedure as the visual analogue scale with a two-sided ruler, but with two reference points added on the plain side in between 0 and 10 cm, at 3.3 cm and 6.6 cm (corresponding to fixed-point scale scores 2 and 3) (Table 1 and Fig. 1).

Satisfactory image quality for evaluating the origin of a coronary artery was set to fixed-point scale 3=visual analogue scale/figurative visual analogue scale 6.6 cm.

### Statistical analysis

Assumption of distribution was performed by using the Shapiro-Wilk test [28]. The results are expressed by mean values, standard deviation (SD) in brackets and 95% confidence interval [CI] using the Student’s *t*-test. Comparisons within and between groups were performed by the paired sample *t*-test and two-sample *t*-test, respectively [29]. Categorical variables are expressed and analysed by contingency tables [30].

Agreement analysis in continuously distributed variables was performed by Bland and Altman plots [31, 32], including estimation of agreement index calculated according to the following formula:

$$ \mathbf{Agreement}\ \mathbf{index}=1-\frac{2{SD}_{\mathbf{difference}\ \mathbf{between}\ \mathbf{observations}}}{\mathbf{mean}\ \mathbf{of}\ \mathbf{observations}} $$

The Bland-Altman plot is used to reveal a relationship between the differences and magnitude of measurements, to look for any systematic bias and (if normality is violated) to identify possible outliers. For categorical variables, weighted kappa analysis was performed [29, 30].

Both agreement index and weighted kappa have the following levels of agreement: <0.20 poor, 0.21–0.40 fair, 0.41–0.60 moderate, 0.61–0.80 good and 0.81–1.00 very good [29].

## Results

The study group consisted of an equal distribution of genders in the transposition of the great arteries group and eight girls and four boys in the control group. The main characteristics of the cohorts are presented in Table 2. All 24 individuals had an SSFP sequence performed and completed the MRI exam without complaints or complications.

**Table 2:** Characteristics of patients with corrected transposition of the great arteries and controls, mean (standard deviation [SD]) and confidence intervals [CI])

	Patients	Controls	Difference
Age in exam year *(years)*	12.0 (1.5)	12.7 (1.7)	0.8 (1.6)
95% CI	11.0–13.0	11.6–13.7	0.12–1.48
Height *(cm)*	151.3 (9.8)	156.3 (13.1)	5.0 (11.5)
95% CI	145.0–157.5	148.0–164.7	0.16–9.83
Weight *(kg)*	41.2 (10.8)	44.5 (13.1)	3.3 (12.0)
95% CI	34.4–48.1	36.2–52.8	−1.7–8.3
Body surface area *(m*^*2*^*)*	1.31 (0.21)	1.38 (0.25)	0.07 (0.23)
95% CI	1.18–1.44	1.22–1.54	−0.03–0.17

The proximal part of the coronary arteries and their ostia were identified in both groups. A few different coronary variants were identified in the patient group and one circumflex artery was not recognized. Additionally, the circumflex artery could not be identified in one healthy volunteer. In these individuals, no left main stem was defined. The result was that 88 coronary origins were identified in the 24 individuals.

**Fig. 2:**
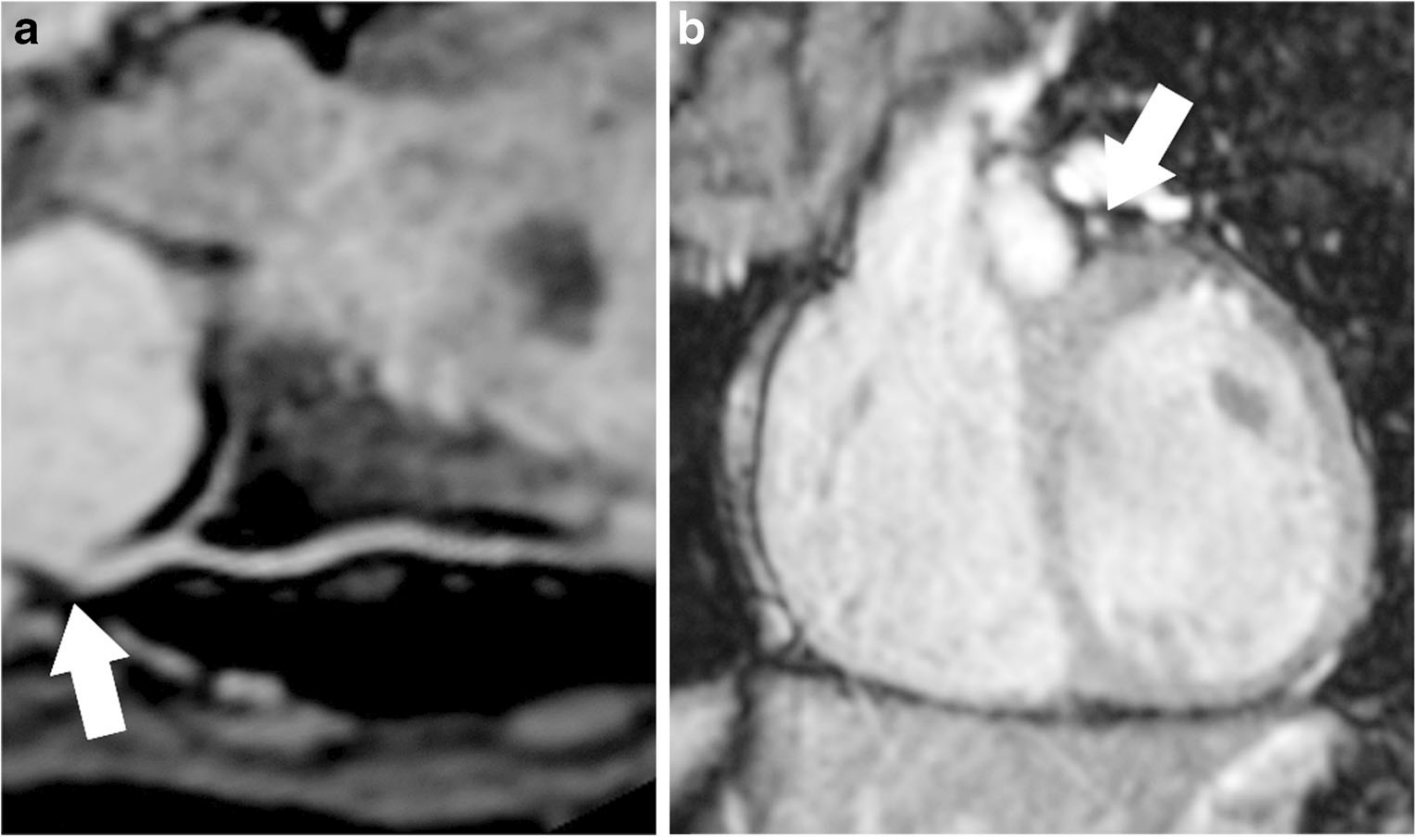
The left main stem (*arrows*). **a**-**b** Multiplanar reconstruction in an aligned plane (**a**) and a perpendicular plane to the origin (**b**) from the steady-state free precession sequence in a patient who had undergone arterial switch operation for transposition of the great arteries. Reader 1’s scoring with the three different methods: fixed-point scale 3, visual analogue scale 7.5 and figurative analogue scale 9.5

The image quality at the origin of the coronary arteries was assessed by the three different methods with results reported in Tables 3, 4 and 5. Figure 2 shows an example with the origin of the left main stem in one individual scored with the three different scoring methods. There was no significant difference in image quality between healthy volunteers and the patients with transposition of the great arteries using the three methods, except in visual analogue scale the first readers’ second reading of the circumflex artery (*P*=0.01) and figurative visual analogue scale second readers’ first reading of the circumflex artery (*P*=0.05) and right coronary artery (*P*=0.03) (Table 5).

**Table 3:** Reader agreement using the fixed-point scale for all subjects

	Intra observer				Interobserver			
Coronary artery	Left main stem	Left anterior descending	Circumflex	Right	Left main stem	Left anterior descending	Circumflex	Right
Mean score	2.8	2.7	2.8	3.3	2.8	2.8	2.5	3.2
κ (standard deviation)	0.52 (0.18)	0.55 (0.01)	0.63 (0.16)	0.77 (0.12)	−0.10 (0.21)	0.35 (0.19)	0.09 (0.12)	0.20 (0.17)
95% confidence interval	0.17–0.87	0.27–0.83	0.31–0.94	0.53–0.99	−0.52-0.32	−0.02-0.72	−0.15-0.34	−0.13-0.54

**Table 4:** Reader agreement using a visual analogue scale (VAS) and a figurative visual analogue scale (fVAS) for evaluating the coronary artery ostia

		Intra-observer				Interobserver			
	Coronary artery	Left main stem	Left anterior descending	Circumflex	Right	Left main stem	Left anterior descending	Circumflex	Right
VAS	Mean score (SD)	4.8 (2.1)	4.4 (2.2)	4.1 (1.8)	6.4 (2.6)	5.7 (2.1)	4.7 (2.0)	4.5 (2.1)	6.3 (2.6)
	Mean difference (SD)	0.6 (2.2)	−0.2 (1.4)	−0.1 (1.6)	0.3 (1.3)	−1.1 (1.8)	−0.9 (1.8)	−0.9 (1.7)	0.3 (1.6)
	Agreement index	0.08	0.35	0.24	0.58	0.37	0.25	0.24	0.50
	Outliers	0/18	3/24	0/22	1/24	1/19	0/21	1/22	0/24
fVAS	Mean score (SD)	6.7 (1.4)	6.8 (1.8)	6.9 (1.7)	8.6 (1.8)	7.0 (1.2)	6.6 (2.0)	6.2 (1.9)	7.7 (2.1)
	Mean difference (SD)	0.6 (1.4)	0.4 (1.9)	0.0 (1.4)	0.0 (1.2)	0.1 (1.2)	0.8 (1.6)	1.4 (1.2)	1.8 (0.7)
	Agreement index	0.59	0.43	0.61	0.71	0.65	0.51	0.61	0.82
	Outliers	1/18	1/24	1/22	1/24	0/18	1/24	1/22	0/24

*SD* standard deviation

**Table 5:** Comparison of image quality of the coronary artery ostia evaluated by reader 1, reading 1 between patients (corrected transposition of the great arteries) and age-matched controls

Coronary artery	Group	*n*	Fixed-point scale				Visual analogue scale		Figurative visual analogue scale	
			1	2	3	4	Mean (SD)	95% confidence interval	Mean (SD)	95% confidence interval
Left main stem	Patients	7	0	2	5	0	4.9 (2.6)	2.5–7.3	7.1 (1.1)	6.0–8.1
	Controls	11	0	4	5	2	5.2 (2.2)	3.7–6.7	7.0 (1.0)	6.3–7.6
Left anterior descending	Patients	12	0	3	8	1	3.9 (2.1)	2.6–5.2	6.6 (1.9)	5.4–7.8
	Controls	12	0	5	7	0	4.6 (2.0)	3.4–5.9	7.3 (1.8)	6.2–8.5
Circumflex	Patients	11	0	4	7	0	3.5 (2.5)	1.8–5.1	6.5 (1.6)	5.3–7.6
	Controls	11	0	2	8	1	4.6 (1.0)	3.9–5.3	7.3 (1.7)	6.2–8.4
Right	Patients	12	0	0	7	5	7.3 (2.3)	5.8–8.7	9.1 (0.9)	8.5–9.7
	Controls	12	0	2	6	4	5.7 (3.0)	3.8–7.6	8.0 (2.4)	6.5–9.5

For qualitative evaluation with a fixed-point scale, a moderate intra observer κ was found for the left main stem (0.52) and left anterior descending artery (0.55) and a good κ value for the circumflex (0.63) and right coronary(0.77) arteries, but there was no positive agreement between the two readers (Table 3).

**Fig. 3:**
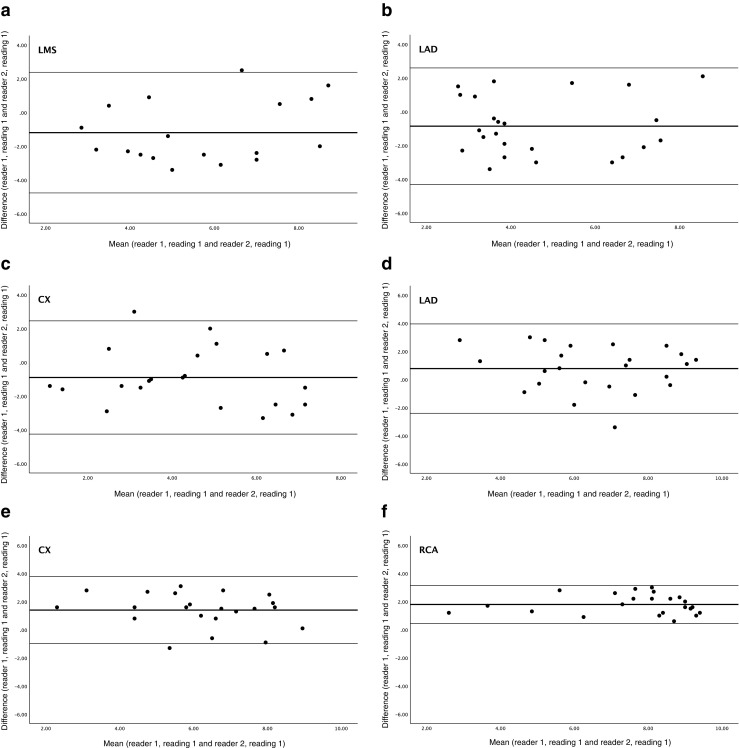
Bland-Altman plot of interobserver agreement. **a-c** With Visual analogue scale for the left main stem (**a**), left anterior descending artery (**b**) and circumflex artery (**c**). **d**-**f** With Figurative visual analogue scale for the left anterior descending artery (**d**), circumflex artery (**e**) and right coronary artery (**f**)

Intra observer agreement index with visual analogue scale was poor to moderate (0.08–0.58), while interobserver agreement index was moderate for the right coronary artery (0.50) (Table 4). The figurative visual analogue scale showed a moderate intra observer agreement index for the left main stem (0.59) and left anterior descending artery (0.43) and good intra observer agreement index for the circumflex (0.61) and right coronary (0.71) arteries, and the interobserver agreement index was good for the left main stem (0.65). The interobserver agreement analysis with visual analogue scale for the left main stem, left anterior descending and circumflex arteries and with the figurative visual analogue scale for the left anterior descending, circumflex and right coronary arteries showed statistically significant difference between the two readers. The agreement index could not be used. However, Bland-Altman plots showed good agreement, but there was a systematic difference between the two readers both when using the visual analogue scale and figurative visual analogue scale for vessel origins (Fig. 3).

## Discussion

In this study, we used three different methods to qualitatively evaluate non-contrast-enhanced 3-T coronary MR angiography image quality. The image quality at the origin of the coronary arteries on SSFP using a fixed-point scale and visual analogue scale was scored with variable reproducibility, while scoring with the figurative visual analogue scale method increased intra- and interobserver agreement.

Image quality is built on two concepts: subjective and objective image quality. The objective evaluation is easier to evaluate in MR, as it consists of quantification of technical parameters, like the contrast-to-noise ratio and signal-to-noise ratio and geometric resolution [33]. Subjective image quality is more challenging. Image quality criteria, like predefined anatomical landmarks with a processing algorithm with different scoring scales, are important in the subjective evaluation made by the radiologist or another observer and are related to his/her opinion and the ability to perceive certain anatomical details. The latter may vary depending on different factors including radiological experience as one of the most important, but also on the surrounding conditions during the assessment and the psychological state of the observer (tired and unfocused versus concentrated and relaxed) [16]. An important issue is to perform a test reading to agree upon the scoring. This is performed on a limited number of cases in consensus as preparation, preceding the main scoring. In this way, the bias of observer understanding and interpretation of the scoring is minimalized.

Subjective image quality should be performed by two or more independent expert readers in a blinded random fashion, but it is known to be a rather time-consuming method.

There are three main approaches proposed to evaluate subjective image quality: the fixed-point scale and the visual analogue scale without and with reference marks.

Image quality in most cases or situations varies along a continuum and can therefore be difficult to determine by strict categorically defined criteria. Using a fixed-point scale may reduce the informative value of the scoring and result in moderate to low intra- and interobserver agreement as determined by the Cohen kappa coefficient test.

In our study, the image quality at the origin of the coronary arteries was assessed using these three different scoring systems. The intra observer agreement was moderate to good for SSFP when using the fixed-point scale, but there was no significant positive agreement between the two readers. In addition, the 95% confidence intervals are large both for intra- and interobserver agreement despite the low sample size emphasizing a great variation. The results with visual analogue scale gave inferior scoring results for intra observer agreement while interobserver agreement was lacking for all coronary arteries except for the right coronary artery. The figurative visual analogue scale gave the same level of intra observer agreement as the fixed-point scale, but the interobserver agreement was very good for the left main stem while lacking for the left anterior descending, circumflex and right coronary artery. This could be explained by the difference in experience between the two readers as there was no significant difference in the two readings made by reader 1 enabling calculation of intra observer agreement index. Having less experience, reader 1 (3 years) had higher scores than reader 2 (10 years), but the Bland-Altman plots showed a systematic difference between readers, substantiating that this could be due to the difference in reader experience (Fig. 3). This difference in rating according to experience was also found in the study by Papanikolaou et al. [18].

Our results could indicate that the qualitative grading of the origin of the coronary arteries is easier and more reliable with the figurative visual analogue scale. Adding the reference points made the scoring easier by giving better differentiation of image quality than the visual analogue scale and fixed-point scale, and one could speculate that using gadolinium-enhanced coronary MR angiography would improve the image quality and further the discrimination of the scoring. Furthermore, considering that clinical studies in paediatric radiology often are restrained to small sample sizes, this method could be important.

We acknowledge some limitations of this methodological pilot study. The sample size is low, which could have the effect that small changes in the scoring of the coronary artery origins in one individual could potentially result in great differences in κ and agreement index.

In the figurative visual analogue scale, the 10-cm scale had reference points that potentially could affect the scorer and change the results.

As a third point, we only evaluated the origin of the coronary arteries, the area with a postoperative risk of kinking and stenosis in patients with corrected transposition of the great arteries. Evaluating the periphery of the arteries would probably be even more challenging in terms of the smaller calibre of the arteries.

## Conclusion

Image quality assessment is particularly important for the much-needed validation of rapidly evolving new imaging techniques. The qualitative assessment is challenging and time-consuming. In this pilot study of coronary MR angiography, the postoperative status of the coronary origin of young patients with corrected transposition of the great arteries was evaluated using three subjective methods; the traditional fixed-point scale compared to the visual analogue scale used in soft data scoring and a modified visual analogue scale version with added reference points. The latter improved both intra- and interobserver agreement.
